# A pilot study of an online behavioral parent training program for children with selective mutism: feasibility and preliminary effectiveness

**DOI:** 10.1186/s13034-025-00976-4

**Published:** 2025-12-24

**Authors:** Tomohisa Yamanaka, Kengo Yuruki, Yoshiaki Koyama, Honami Koyama, Masahiko Inoue

**Affiliations:** 1 Student Accessibility Office, Head Office for Education and Student Support, Shimane University, Matsue, 690-8504 Japan; 2 Tottori Prefecture Welfare Counseling Center, Tottori, 680-0901 Japan; 3 Department of Doctoral Course, Graduate School of Medical Sciences, Tottori University, Yonago, 683-8503 Japan; 4 Yonago Child Welfare Center, Yonago, 683-8503 Japan; 5 Academic Research Institute Faculty of Education, Shimane University, Matsue, 690- 8504 Japan; 6 Department of Clinical Psychology, Graduate School of Medical Sciences, Tottori University, 86 Nishi-cho, Yonago, 683-8503 Japan

**Keywords:** Selective mutism, Behavioral parent training, Developmental disorders, Online intervention, Feasibility study

## Abstract

**Background:**

Recent approaches to treating selective mutism (SM) have increasingly emphasized parent involvement, and several parent-mediated programs have shown promising efficacy. However, in regions where SM specialists are scarce, families have limited access to appropriate support. In this context, online behavioral parent training (BPT) may serve as a promising and scalable option that can overcome geographic barriers. Nevertheless, empirical studies specifically targeting SM-focused BPT remain limited. This pilot study evaluated the feasibility and preliminary effectiveness of an online BPT program developed for parents of children with SM.

**Methods:**

This pilot study evaluated the feasibility and preliminary effectiveness of a nine-session online behavioral parent training program (SM-BPT) for parents of children aged 3 to 9 years with diagnosed or suspected SM. Seventeen parents enrolled in the program, and 13 completed both the intervention and the pre- and post-assessments. Changes in children’s SM symptoms, anxiety, and behavioral problems were assessed alongside parenting behaviors and parental mental health.

**Results:**

Following the intervention, significant improvements were observed in children’s school-based SM symptoms (*p* = .04), anxiety (*p* = .03), and internalizing (*p* = .03) and externalizing behaviors (*p* = .02). However, no significant changes were found in total SM symptoms (*p* = .22) or home/family communication (*p* = .83). Parents reported reduced negative parenting behaviors (*p* < .001) and improved mental health (*p* < .01). In contrast, positive parenting did not significantly change (*p* = .72). High attendance (mean = 93.5%) and homework completion (mean = 73.5%) supported the program’s feasibility and acceptability.

**Conclusions:**

The SM-BPT demonstrated preliminary effectiveness in reducing children’s SM symptoms at school, as well as anxiety and behavioral problems, and in improving parental mental health. High attendance rates also suggested good feasibility. Because the program can be delivered online, it may serve as a practical option for families with limited access to specialized support. Further research, including randomized controlled trials, is needed to examine its effectiveness across diverse family situations.

*Trial registration* UMIN Clinical Trials Registry UMIN000043686, registered on 21 March 2021

## Background

Selective mutism (SM) is an anxiety disorder characterized by a consistent failure to speak in specific social settings, despite having normal language abilities [1]. SM is closely linked to social anxiety with comorbidities consistently reported across studies [2]. Autism spectrum disorder (ASD) is listed as a diagnostic exclusion for SM in the Diagnostic and Statistical Manual of Mental Disorders, Fifth Edition, Text Revision (DSM-5-TR). However, some children with SM present with co-occurring conditions such as ASD [3, 4]. Despite these observations, empirical research on the comorbidity and phenotypic overlap between SM and ASD remains limited, underscoring the need for further investigation. Individuals with a history of SM are at increased risk of long-term interpersonal challenges and psychiatric vulnerability [4, 5], emphasizing the importance of early identification and intervention.

Several studies have suggested that cognitive behavioral therapy may benefit children with SM [6], using techniques such as contingency management, shaping, graduated exposure, and stimulus fading [7]. Parental involvement is crucial in early-stage SM interventions, as children often avoid direct interactions with clinicians [8]. However, some parental behaviors may unintentionally sustain symptoms through negative reinforcement [9]. Recent studies have shown that while parental accommodation is relatively common in families of children with SM, its frequency is often underreported in self-administered questionnaires, and parents tend not to perceive such behaviors as sources of stress [10]. This discrepancy may reflect limitations in measurement tools or parents’ awareness of their behaviors [10]. Therefore, future parent-focused interventions should adopt a more nuanced understanding of the role of these behaviors and appropriately position them within treatment strategies.

Recently, increasing attention has been paid to parent-based interventions for children with SM, including the Parent-Child Interaction Therapy for Selective Mutism (PCIT-SM), Intensive Group Behavioral Treatment for Selective Mutism (IGBT-SM), and Specific Parenting for Anxious Childhood Emotions adapted for SM (SPACE-SM). The PCIT-SM aims to reduce parental behaviors that reinforce children’s avoidance and promote verbal communication in children with SM [11]. In a 16-session trial, 58.6% of children were classified as robust responders, which indicated clinically meaningful, although partial, improvements in SM symptoms [11]. The IGBT-SM is a brief, group-based intervention that incorporates elements from the PCIT-SM, delivered intensively over one week [12]. In a waitlist-controlled trial, 50% of the participants responded to treatment, and 46% no longer met the SM criteria at the eight-week follow-up [13]. Aldrich et al. assessed an eight-week PCIT-SM–based group program for children aged 3–14 years and their parents, beginning with a parent-only session followed by seven joint sessions [14]. Results revealed reduced SM symptoms, increased verbal communication, and improved parental behavioral support. However, the effects on parental mental health and stress were limited, suggesting additional parent-focused support. In contrast, the SPACE-SM is a parent-focused intervention that targets family accommodation, how parents temporarily relieve their child’s anxiety, rather than the child’s anxiety symptoms themselves [15, 16]. Tse conducted a pilot non-inferiority trial that compared SPACE-SM and IGBT-SM in 10 families of children with SM aged 4–10 years (*n* = 4 and *n* = 6, respectively) and found 50% remission in both groups [15]. Pabis evaluated the SPACE-SM delivered via synchronous videoconferencing in a randomized trial (intervention group, *n* = 26; waitlist, *n* = 27) [16]. Although improvements in children’s verbal behavior were limited, reduced parental accommodation and anxiety were observed.

These interventions have significantly expanded treatment options for SM. However, in both the PCIT-SM and IGBT-SM, the parents played a supplementary role, while trained clinicians delivered the main therapeutic elements. Therefore, these programs may be difficult to implement in regions with limited access to specialized services, which can lead to delays in treatment initiation. A remote version of the IGBT-SM has been piloted, with promising preliminary results; however, concerns remain regarding reduced opportunities for individualized support, such as scaffolding and reinforcement [17]. While SPACE-SM offers geographically accessible support via remote delivery, it does not explicitly target SM symptoms or verbal behaviors, which may limit its suitability for early interventions that require structured, stepwise behavioral support. Furthermore, few existing interventions have been adapted for children with SM and comorbid developmental disorders, such as ASD. Given these limitations, developing interventions that (1) are feasible in low-resource settings, (2) can be delivered during early developmental stages, and (3) are adaptable for children with comorbid developmental conditions, such as ASD, is essential.

Behavioral parent training (BPT) is a promising approach and widely implemented intervention that improves children’s behavioral difficulties through parental involvement. BPT teaches behaviorally grounded strategies to enhance child outcomes and parental mental health, and has well-established efficacy, particularly for children with developmental disorders [18]. Notably, BPT can be delivered online, making it accessible to families in underserved regions [19, 20]. Its parent-mediated model also makes it well-suited for early intervention in infancy and preschool years when timely support is critical.

However, the application of BPT to children with SM remains limited. A recent case study [21] reported on an adapted BPT program, originally developed for children with developmental disorders, that was modified for children with SM and delivered online to the parents of a child with SM. The program included techniques in applied behavior analysis , environmental modifications, and gradual exposure, aiming to reduce the child’s anxiety through parent-mediated strategies. While elements such as Antecedent-Behavior-Consequence analysis (ABC analysis), positive reinforcement, and environmental modification are commonly used in interventions for children with ASD and developmental delays, they were selected here for their relevance to anxiety and avoidance behaviors associated with SM, rather than for any diagnostic specificity. The parent implemented these strategies both at home and in community settings, resulting in improvements in the child’s SM symptoms and a reduction in parental stress. These findings support the feasibility of delivering SM-specific BPT online, though further research is needed to evaluate its effectiveness in larger and more diverse samples.

In this pilot study, we examined the preliminary effectiveness of an SM-specific behavioral parent training program (SM-BPT), adapted from a previous case-based intervention by Yamanaka et al. [21]. This program was delivered online to several parents of children with SM. We used a pre-post design and assessed two hypotheses: (1) the intervention would reduce children’s SM symptoms, anxiety, and behavioral difficulties by equipping parents with strategies to manage their children’s anxiety, and (2) the intervention would improve parental mental health, reduce parenting stress, and enhance parenting practices.

## Methods

### Eligibility criteria and participant recruitment procedures

Participants were recruited via flyers posted on the last author’s website, at a university hospital, and at university-affiliated counseling centers. Eligibility criteria included being a parent aged ≥ 20 years with a child aged 3–9 years who had not spoken in specific social settings (e.g., kindergarten, school) for at least one month. This criterion was designed to reflect the DSM-5-TR definition of SM [1]. According to the DSM-5-TR [1], SM is characterized by a consistent failure to speak in specific social situations despite speaking in other settings, such as at home, indicating that total silence across all contexts is not required. In line with this definition, the present study included children whose speaking behavior varied depending on the social context (e.g., school) and interaction partners (e.g., teachers, peers). A stable internet connection and basic technical skills were also required.

Of the 24 individuals who expressed interest, 17 met the eligibility criteria and were enrolled in the program. Among them, 13 participants were included in the final analysis. Four participants were excluded from the analysis due to missing outcome data (i.e., although they participated in the program, their post-intervention questionnaires were not returned by the deadline).

Informed consent was obtained after a study explanation was provided via Zoom. Demographic data for the 17 enrolled participants (e.g., child’s diagnosis, use of special education services, household income) were collected via an online form. Diagnostic and suspected diagnostic information, including SM, ASD, attention-deficit/hyperactivity disorder (ADHD), and intellectual disability (ID), was obtained via parent report without independent verification. A “suspected diagnosis” denoted instances where healthcare, psychological, or educational professionals expressed concerns or made informal comments suggesting a potential condition in the absence of a formal diagnostic evaluation.

### Training staff

The program was delivered by a multidisciplinary team comprising a main facilitator for lectures, group facilitators, a technical support manager, and a supervisor. Team members included six clinical psychology master’s students, one doctoral student, four special needs school teachers, and one university faculty member specializing in behavioral therapy.

### Selective mutism behavioral parent training

The SM-BPT program consisted of nine bi-weekly, two-hour sessions (Table 1), structured around six core themes: (1) praise strategies, (2) ABC analysis, (3) environmental modifications, (4) verbal prompting in anxiety-inducing situations, (5) step-by-step exposure hierarchies, and (6) caregiver responses to behavioral challenges. Each session included lectures and group work, with homework reviews starting from Session 2. Sessions incorporated 2–3 group discussions (10–20 min each) to deepen understanding through participant–staff interaction.

The program was adapted from a previous case-based version by Yamanaka et al. [21], PCIT-SM [11], and an online BPT for developmental disorders [18]. Mindfulness elements, such as stress-related psychoeducation and guided meditation, were also included.

**Fig. 1:**
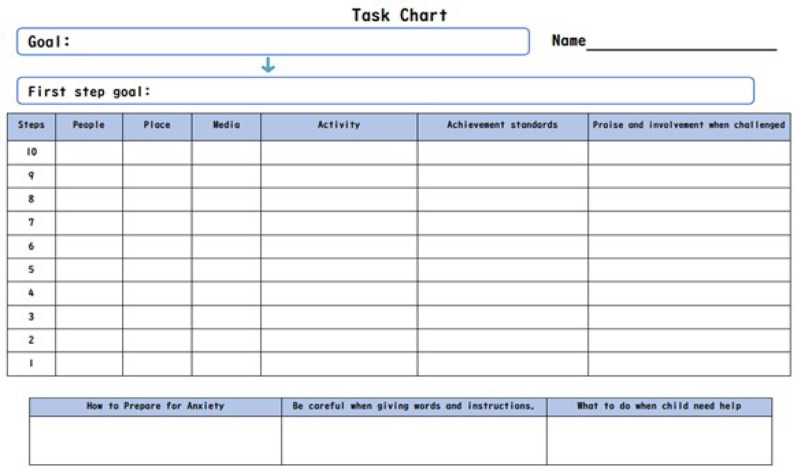
Task chart

Each session had a clear theme: Session 1 introduced SM and the program; Sessions 2–3 covered praise, reinforcement, and ABC analysis; Sessions 4–6 focused on environmental adjustments, prompting, and the development of an individualized task chart to guide gradual exposure (Fig. 1). Sessions 7–8 addressed implementation issues and refinements; Session 9 involved sharing of outcomes and psychoeducation on long-term support. Participants used nicknames for anonymity and joined a closed social media group for questions and support. Staff monitored the group to ensure safety.

Homework assignments were provided at the end of each session, and participants were instructed to submit them via email before the start of the following session. When task chart creation or implementation was included as a homework assignment (e.g., from Session 6 onward), the same submission procedure applied; in some cases, progress on task charts was also reviewed during the subsequent online session. Homework completion and task chart usage were recorded based on parent submissions and facilitator documentation.

**Table 1:** Contents of selective mutism behavioral parent training

Session	Content	Group Work	Homework
1	• Orientation • Psycho-education of SM • Points on how to praise well	• Think of a game to play during a special time	1. Create a special time
2	• How to praise children with SM according to their characteristics	• Homework sharing • Sharing of everyday praise for the patient • Thinking about the best praise words to use for children • Mindfulness	1. Practice praising that makes children happy. 2. Record children’s responses to praise
3	• Mechanism by which SM is maintained • ABC analysis,	• Homework sharing • Analyzing the circumstances under which the patient caused SM symptoms using ABC analysis • Mindfulness	1. Practice ABC analysis using a fictional SM case
4	• Environmental adjustments to elicit adaptive behavior	• Homework sharing • Considering environmental adjustment methods • Mindfulness	1. Practice environmental adjustment using a fictional SM case
5	• Communication methods that elicit adaptive behavior according to SM characteristics	• Homework sharing • Examine how to talk to and interact with children with SM when their anxiety is heightened • Create small steps for the patient, list the degree of speech with people, places, media, and activities • Mindfulness	1. Add or revise the items on the list and fill in the degree of speech
6	• How to construct small steps • Devices to assist children with SM when practicing small steps	• Homework sharing • Determine the goals you want your child to achieve • Fill in a “task chart” with small steps using a list • Mindfulness	1. Practice task chart created in this session 2. Record the practice results
7	• How to deal with problems caused by the child’s SM symptoms when practicing small steps	• Homework sharing • Preparing a task chart • Mindfulness	1. Practice the task chart 2. Record the practice results
8	• A comprehensive review of the program content	• Homework sharing • Preparing a task chart • Mindfulness	1. Practice the task chart 2. Record the practice results
9	• Prognosis and future of children with SM • Completion ceremony	• Homework sharing • Share your thoughts regarding your participation in SM-BPT • Mindfulness	

ABC = Antecedent–Behavior–Consequence; SM = Selective mutism; SM-BPT = Behavioral parent training developed for SM

### Study design

This pre-post study, without a control group, examined the effectiveness of the SM-BPT delivered to parents of children with SM. Participants completed psychological assessments pre- and post-intervention.

### Measurements

#### Selective mutism questionnaire Japanese version (SMQ-JP)

The SMQ-JP was a Japanese adaptation of the Selective Mutism Questionnaire (SMQ; [22]), developed by Kanmoku Net [23]. It included 17 items that assessed SM severity across three domains: school, home, and public. Each item was rated on a 4-point scale (0 = *never* to 3 = *always*), and higher scores indicated fewer speaking difficulties. Although the original version had good reliability and validity, the Japanese version has not been fully validated. In our study, the Cronbach’s alpha of the SMQ-JP, calculated from pre-intervention data, was 0.80. Both total and subscale scores were used to assess intervention-related changes. In this study, both total and subscale scores were converted to mean item scores to allow comparability with previous studies.

To further aid interpretation, a previous study reported baseline mean item scores on the original SMQ of 0.87 (SD = 0.36) for children with SM (*n* = 32), and 2.58 (SD = 0.23) for typically developing children (*n* = 32) [24].

#### Young children’s anxiety tendencies scale (YCATS)

The YCATS was a 28-item parent-report measure developed by Nishizawa to assess general anxiety tendencies during early childhood [25]. It demonstrated adequate internal consistency, concurrent validity, and face validity. In our study, the Cronbach’s alpha of the YCATS, calculated from pre-intervention data, was 0.80. Items reflected anxiety-related behaviors, such as “rarely seen talking with peers other than close friends,” "appears nervous or anxious during group activities in class", and "reluctant to separate from caregiver in unfamiliar settings". Each item was rated on a 6-point scale (0 = *not at all* true to 5 = *true*), and higher scores indicated greater anxiety tendencies. The total score was used to assess changes post-intervention.

#### Child behavior check list 4–18 Japanese version (CBCL)

The CBCL, developed by Achenbach, was a 120-item parent-reported scale that assessed behavioral and emotional problems in children [26]. This study used the Japanese version, which consists of 113 items and has demonstrated high reliability and validity [27]. In our study, the Cronbach’s alpha of the CBCL, calculated from pre-intervention data, was 0.95. It comprised three domains: internalizing, externalizing, and total problems. Items were rated on a 3-point scale (0 = not true to 2 = very true or often true), and higher scores indicated greater symptom severity. Raw scores were converted to age-standardized T-scores (mean = 50, SD = 10). T-scores for the three domains were used to assess intervention-related changes.

#### Parent stress index Japanese version (PSI)

The PSI was a 101-item measure developed by Abidin et al. to assess parenting stress related to parent–child and family functioning [28]. This study used the Japanese version, which consists of 78 items and has demonstrated high internal consistency, content validity, and concurrent validity [29, 30]. In our study, the Cronbach’s alpha of the PSI, calculated from pre-intervention data, was 0.93. It included two subscales: the Parent Domains (e.g., restriction of role and sense of competence) and Child Domains (e.g. distractibility/hyperactivity and reinforcement of parent). Items were rated on a 5-point Likert scale (1 = *strongly disagree* to 5 = *strongly agree*), and higher scores indicated greater parenting stress. The total score was used to assess intervention-related changes.

#### Positive and negative parenting scale (PNPS)

The PNPS was a 24-item parent-report measure developed by the PNPS Development Team to assess both positive and negative parenting behaviors [31]. It demonstrated acceptable reliability and construct validity. In our study, the Cronbach’s alpha of the PNPS, calculated from pre-intervention data, was 0.73. Items included “I try to respect my child’s wishes as much as possible,” “I say thank you when my child does something helpful,” and “I sometimes take out my frustrations on my child.” Each item was rated on a 4-point scale (1 = *rarely* or *never* to 4 = *very frequently*). Higher and lower scores on the positive and negative subscales indicated more desirable parenting practices, respectively. Both scores were used to evaluate intervention-related changes.

#### 30-Item general health questionnaire 30 Japanese version (GHQ-30)

The GHQ-30, developed by Goldberg et al., assessed mental and physical health over the past 2–3 weeks across six domains: general disease tendency, physical symptoms, sleeplessness, social activity disorder, depressive tendency, and anxiety/caprice [32]. This study used the validated Japanese version [33], which demonstrated high reliability and validity. In our study, the Cronbach’s alpha of the GHQ-30, calculated from pre-intervention data, was 0.98. Higher total scores indicated poorer mental health. The total score was used to assess intervention-related changes.

### Statistical analysis

Descriptive data on session attendance, homework completion, and task chart usage were compiled for all 17 participants. Statistical analyses were conducted on data from 13 participants who attended at least six SM-BPT sessions and submitted post-intervention measures within the designated period. The Shapiro–Wilk test was used to assess normality. Paired t-tests and Wilcoxon signed-rank tests were applied to normally distributed and non-normally distributed variables, respectively. Statistical significance was set at *p* <.05. Since this study was exploratory, corrections for multiple comparisons were not applied. Effect sizes (r) were calculated and interpreted as small (0.10), medium (0.30), or large (0.50) [34]. Statistical analyses were performed using IBM SPSS version 25.0.

## Results

### Participants’ demographic characteristics

Tables 2 and 3 present the parents’ and children’s characteristics. Children had a mean age of 6.47 years (SD = 1.45; range: 4–9), with four boys (23.5%) and 13 girls (76.5%). Of these, 10(58.8%) had a formal SM diagnosis, while seven(41.1%) were suspected cases. Furthermore, six (35.3%) had ASD diagnoses, and three (17.6%) were suspected. Additionally, three (17.6%) had ADHD, one (5.9%) had a specific learning disorder (SLD), and one(5.9%) had an intellectual disability(ID), while two (11.8%) were suspected of having an ID. Five children (29.4%) did not attend school, and 15 (88.2%) regularly used the hospital or clinic services.

Participating parents had a mean age of 40.64 years (SD = 4.83), and all were mothers. Furthermore, seven (41.2%) were employed, seven were not, and three (17.6%) did not respond. Regarding education, three (17.6%) completed high school, five (29.4%) held university degrees, two (11.8%) held graduate degrees, and seven (41.2%) had other backgrounds. Annual household income most commonly fell within the 3–4-million-yen range (*n* = 4), followed by 5–6 and ≥ 10 million yen (*n* = 3 each).

**Table 2:** Participants’ profiles

ID	Age	Sex	Last educational background	Employment situation	Number of attendances	Number of homework submissions	Achievement of small steps
1	37	F	High School	Employed	9/9(100.0%)	8/8(100.0%)	Fully achieved
2	43	F	High School	Unemployed	8/9(87.5%)	8/8(100.0%)	Fully achieved
3	38	F	Graduate School	Employed	9/9(100.0%)	8/8(100.0%)	Achieved approximately half
4	40	F	Specialized Training College	Employed	9/9(100.0%)	3/8(37.5%)	Achieved some steps
5	36	F	Junior college	Unemployed	9/9(100.0%)	8/8(100.0%)	Fully achieved
6	41	F	University	Unemployed	9/9(100.0%)	8/8(100.0%)	Fully achieved
7	37	F	Graduate School	Employed	9/9(100.0%)	8/8(100.0%)	Fully achieved
8	41	F	Specialized Training College	Employed	9/9(100.0%)	8/8(100.0%)	Fully achieved
9	50	F	Junior college	No response	8/9(87.5%)	3/8(37.5%)	Achieved some steps
10	45	F	University	Unemployed	8/9(87.5%)	8/8(100.0%)	Fully achieved
11	48	F	Junior college	Unemployed	6/9(75.0%)	4/8(50.0%)	Achieved some steps
12	37	F	University	Unemployed	9/9(100.0%)	7/8(87.5%)	Achieved approximately half
13	37	F	University	Employed	9/9(100.0%)	2/8(25.0%)	Achieved some steps
14*	30	F	High School	No response	8/9(87.5.%)	2/8(25.0%)	Achieved some steps
15*	42	F	Specialized Training College	Employed	9/9(100.0%)	6/8(75.0%)	Fully achieved
16*	46	F	Junior college	Unemployed	8/9(87.5%)	7/8(87.5%)	Fully achieved
17*	43	F	University	No response	7/9(77.77%)	2/8(25.0%)	Achieved some steps

F = Female; * = Participants excluded from analysis

**Table 3:** Profile of the participants’ children

ID	Age	Sex	Primary Diagnosis	Other Diagnoses	Child’s school attendance
1	6	M	SM		Preschool
2	5	F	Suspected SM		Kindergarten
3	6	F	SM	ASD	Kindergarten
4	7	F	Suspected SM		Elementary school(Regular class)
5	5	F	SM		Kindergarten
6	4	F	Suspected SM		Kindergarten
7	6	F	SM	ASD ADHD Suspected ID	Elementary school (Special support education class)
8	6	F	SM		Kindergarten
9	8	F	Suspected SM	SLD Suspected ASD	Elementary school (Regular class) (SNA)
10	5	F	SM	ASD	Kindergarten
11	7	M	SM	ASD ADHD	Elementary school (Regular class) (SNA)
12	5	M	SM		Preschool
13	9	F	Suspected SM	ASD	Elementary school (Regular class) (SNA)
14*	6	F	Suspected SM		Elementary school (Regular class)
15*	5	F	SM	Suspected ASD Suspected ID	Kindergarten
16*	8	M	SM	ASD ADHD ID	Elementary school (Special support education class) (Truancy)
17*	9	F	Suspected SM	Suspected ASD	Elementary school (Regular class) (SNA)

F = Female; M = Male; SM = selective mutism; ASD = autism spectrum disorder; ADHD = attention-deficit/hyperactivity disorder; ID = intellectual disability; SLD = specific learning disorder; SNA=school non-attendance. *Children excluded from analysis

### Demographic characteristics of the analyzed parents and children

The 13 analyzed parents had a mean age of 40.76 years (SD = 4.37; range: 30–50). Of these, two (15.3%) had completed high school, two specialized training sessions, three junior colleges, four universities, and two graduate schools. Annual income was most commonly within the 5–6 and ≥ 10-million-yen range (*n* = 3 each), followed by 3–4 and 6–7 million (*n* = 2 each), and 7–8, 8–9, and 9–10 million yen (*n* = 1 each).

This study included 13 children whose parents completed at least six SM-BPT sessions and submitted post-intervention data. Their mean age was 6.30 years (SD = 1.38; range: 4–9), with three boys (23.1%) and 10 girls (76.9%). Eight(61.5) had diagnosed SM, and five(38.4%) were suspected. Furthermore, five had ASD, one was suspected, two had ADHD, and one had SLD. Eleven (84.6%) received regular support, and three (23.1%) did not attend school.

As shown in Tables 2 and 3, the demographic characteristics of the four participants excluded from analysis were generally comparable to those of the 13 participants included in the final analysis.

### Program attendance

Session attendance rates ranged from 88.2 to 100%, with an average of 93.4%. Of the 17 participants, 12 attended all sessions, five missed one, one missed two, and one missed three. Attendance was 100% for sessions 1 and 8, 94.1% for 2, 4, 5, and 6, and 88.2% for 3, 7, and 9.

### Homework completion and task chart implementation

Regarding homework submission rates, sessions 1, 2, 3, 4, and 5–8 had 100%, 94.1%, 82.3%, 52.9%, and 64.7%, respectively. The average submission rate was 73.5%. Eight participants (47.1%) submitted all the assignments, two submitted 87.5%, one submitted 75.0%, one submitted 50.0%, two submitted 37.5%, and three submitted 25.0%. Regarding task chart implementation, eight participants completed all the steps, three completed approximately half, and six implemented steps on two to three occasions.

### Normality testing

Shapiro–Wilk tests were used to assess normality pre- and post-intervention. Normality was confirmed for the SMQ-JP total and “home/family” subscale, CBCL total and internalizing subscales, and PSI total. Paired t-tests were used for analyses. Non-normal distributions were found for the remaining seven measures, which included the other SMQ-JP subscales: YCATS, CBCL externalizing, GHQ-30, and PNPS positive and negative. The Wilcoxon signed-rank test was performed.

### Changes in parent measures

Pre-post changes in the PSI, GHQ-30, and PNPS were analyzed using paired t-tests or Wilcoxon signed-rank tests (Tables 4 and 5). The GHQ-30 scores significantly decreased (*z* = −2.62, *p* <.01, *r* =.73) while PSI scores did not change significantly (*t*(12) = −2.07, *p* =.06, *r* =.51). Negative parenting demonstrated significant improvement (*z* = −2.82, *p* <.01, *r* =.79), whereas positive parenting demonstrated no significant change (*z* = −0.35, *p* =.72, *r* =.10).

### Changes in child measures

Changes in the SMQ-JP, YCATS, and CBCL scores were examined via paired t-tests or Wilcoxon signed-rank tests (Tables 4 and 5). No significant changes were observed in the SMQ-JP total (*t*(12)=−1.26, *p* =.22, *r* =.34), home/family (*t*(12) = −0.21, *p* =.83, *r* =.06), or social situations subscales (*z* = −0.06, *p* =.95, *r* =.02). Significant improvements were observed in the kindergarten/school subscale (*z*=−2.04, *p* =.04, *r* =.57) and YCATS (*z*=−2.09, *p* =.03, *r* =.58). The CBCL scores significantly improved for total (*t*(12) = 2.98, *p* <.01, *r* =.65), internalizing (*t*(12) = 2.46, *p* =.03, *r* =.58), and externalizing problems (*z* = −2.27, *p* =.02, *r* =.63).

**Table 4:** Mean and standard deviation of the SMQ-JP (total, home/family), CBCL (total, internalizing), and PSI (*N* = 13)

Outcome measure		Pre			Post			t	*p*	Effect size *r*
		Mean	SD		Mean	SD				
Child	SMQ-JP-total	0.76	0.46		0.86	0.52		−1.269	0.22	0.34
	SMQ-JP-home/family	1.44	0.80		1.47	0.77		−0.215	0.83	0.06
	CBCL T-score total	56.07	8.80		53.23	10.62		2.986	0.00	0.65
	CBCL internalizing T-score	70.00	11.55		67.00	11.05		2.460	0.03	0.58
Parent	PSI total	226.15	35.76		217.61	40.2		2.079	0.06	0.51

**Table 5:** Median and interquartile range of the SMQ-JP (kindergarten/school, public), YCATS, CBCL (externalizing), GHQ-30, and PNPS (positive, negative) (*N* = 13)

Outcome measure		Pre					Post				Z	*p*	Effect size *r*
		Median	Interquartile range				Median	Interquartile range					
Child	SMQ-JP-kindergarten/school	0.16	0.00	–	2.00		0.66	0.00	–	2.16	−2.047	0.04	0.57
	SMQ-JP-public	0.47	0.00	–	1.40		0.47	0.00	–	1.40	−0.061	0.95	0.02
	YCATS	112.00	105.00	–	115.00		102.00	97.00	–	108.00	−2.098	0.03	0.58
	CBCL externalizing T-score	54.00	49.00	–	57.00		46.00	42.00	–	58.00	−2.271	0.02	0.63
Parent	GHQ-30	8.00	6.00	–	11.00		1.00	0.00	–	6.00	−2.626	0.00	0.73
	PNPS-Positive	36.00	35.00	–	41.00		39.00	36.00	–	40.00	−0.354	0.72	0.10
	PNPS-Negative	20.00	19.00	–	22.00		19.00	17.00	–	20.00	−2.829	0.00	0.79

## Discussion

This study was the first to apply the SM-BPT, previously reported only in single-case studies, to a parent group. Using a pre-post design, we examined whether online SM-BPT improved SM symptoms in children as well as parental mental health. The results revealed significant improvements in parental mental health and negative parenting and children’s mutism severity (kindergarten/school), anxiety, and internalizing/externalizing behaviors. These findings supported and partially supported the first and second hypotheses, respectively.

### Feasibility and acceptability of SM-BPT

The SM-BPT helped parents support their children with SM through environmental adjustment and exposure strategies. No participants dropped out, and the average session attendance was 93.5%, higher than in previous online BPT studies [19, 20], which suggested low participant burden. Accessibility via the online format was a contributing factor. In addition, scheduling evening sessions may have made participation easier for working parents.

Homework completion averaged 73.5%, which exceeded that of prior reports. However, a decline was noted from Session 4, possibly due to increasing task difficulty and time constraints. These results suggested the need to refine the program structure, such as by adjusting task difficulty and initiating small step planning earlier.

### Impact on parental mental health and parenting behaviors

This study observed improved mental health and reduced negative parenting behaviors. In contrast, no significant changes were observed in positive parenting behaviors (*r* =.10). Previous research indicated that enhanced parental mental health may be associated with improvements in children’s adaptive abilities [18]. Reductions in child difficulties may have contributed to improved parental mental health. A significant effect was observed for the GHQ-30 (*r* =.73). Although the PSI did not reach statistical significance, it demonstrated a large effect size (*r* =.51). These improvements also reflected the benefits of peer support through group sessions and online communication.

Since the SM-BPT focused on anxiety reduction and minimizing maladaptive responses, parents were encouraged to adopt new interaction patterns with their children, which may have decreased negative parenting behaviors. The baseline mean score for positive parenting was 36 out of 48, which suggested participants were already engaged in relatively constructive practices. Although this does not imply a ceiling effect, a high initial score may limit the room for observable changes.

However, since this study employed a single-group pre-post design without a control group, the findings should be interpreted with caution. While the observed improvements are promising, future research using randomized controlled trials or waitlist-controlled designs should clarify the causal impact of the SM-BPT.

### Impact on children’s SM symptoms and behaviors

Parental efforts to implement environmental adjustments and gradual exposure strategies may have influenced improvements in children’s SM symptoms and behavioral functioning. These interventions likely reduced anxiety-provoking stimuli and facilitated communication among the children. In addition, reduced negative parenting behaviors may have disrupted the patterns of negative reinforcement that previously maintained SM behaviors, which promoted further adaptive functioning.

Observed improvements in externalizing problems suggested that this program, which shared core features with conventional BPT, was effective for children with co-occurring or suspected developmental disorders. These findings underscore the potential utility of the SM-BPT in various clinical settings, particularly those where SM coexists with other developmental conditions.

### Implications for practice and implementation

These findings suggest that SM-BPT may foster parental engagement and improve children’s SM symptoms, anxiety, and behavioral difficulties. Notably, improvements in internalizing and externalizing problems—including among children with co-occurring or suspected developmental disorders—indicate its applicability to families with more complex needs. While most SM intervention programs developed to date involve parent–child or child-focused sessions, few parent-only approaches have demonstrated measurable benefits. The remote, parent-focused group format enhances accessibility and feasibility. Moreover, prior research shows that parents of children with SM seek opportunities to gain knowledge, consult professionals, and access concrete strategies [35], all of which are addressed by SM-BPT. Dissemination at the community level may help bridge service gaps and reduce barriers to care. Future efforts should aim to establish SM-BPT as a sustainable model across educational and healthcare systems.

To support implementation, future studies should incorporate follow-up assessments to evaluate delayed intervention effects on child-related outcomes. Evidence shows that the full impact of parent training programs often emerges gradually, as parents begin to consistently implement new strategies in their daily routines [36]. Follow-up evaluations can clarify the persistence and developmental trajectory of these effects.

### Strengths and limitations

This study is the first to evaluate the feasibility and preliminary utility of a remotely delivered group-based BPT program for parents of children with SM. Strengths include the program’s capacity to facilitate positive behavioral changes in both children and parents and its applicability to children with co-occurring developmental disorders. Remote delivery also enhances accessibility for underserved families.

However, this study has several limitations. First, the small sample size (*n* = 13), absence of a control group, and use of a single-group pre-post design limited causal inference and generalizability. Second, multiple statistical tests were conducted without correction, which increased the risk of Type I errors. Since this was an exploratory study, future research should use larger samples, randomized controlled designs, and appropriate statistical adjustments. Third, while some children had received a formal SM diagnosis from a medical institution, diagnostic status was based on parent report, and not all participants had a confirmed diagnosis. Additionally, the research team did not conduct clinical interviews to confirm the diagnosis of SM or to assess comorbid conditions such as ASD. This reliance on parent-reported diagnoses and online recruitment may have introduced sampling bias and the risk of false positives. Future studies should include participants with professionally confirmed diagnoses and consider recruitment through clinical settings such as hospitals, child psychiatry departments, or developmental outpatient clinics where formal diagnoses are made by qualified specialists (e.g., child psychiatrists).

Fourth, all outcome measures were based on parent reports. Additionally, the SMQ-JP, which was used as the primary outcome measure, has not been fully validated in the Japanese context. Such limitations are likely to reduce the interpretability and generalizability of the findings. Furthermore, the PNPS showed a somewhat lower internal consistency (α = 0.73). Future studies should employ psychometrically robust measures and incorporate behavioral or observational assessments to enhance validity. Lastly, program fidelity was not assessed. Monitoring adherence to intervention protocols is essential for evaluating the program’s effectiveness.

## Conclusion

This study explored the preliminary utility of SM-BPT for parents of children with SM using a pre-post design. Improvements were observed in parental mental health, parenting behaviors, and children’s SM symptoms, anxiety, and behavioral problems. Most parents successfully implemented exposure tasks, suggesting that parent-mediated strategies can support behavioral change in children. SM-BPT appears to be a promising, adaptable option, especially for families in underserved settings or with co-occurring conditions. However, conclusions are limited by the lack of a control group; future RCTs with larger samples are needed to confirm its effectiveness.

## Data Availability

The data supporting the findings of this study are not publicly available due to participant confidentiality. De-identified data may be made available from the corresponding author upon reasonable request and may require additional ethics approval.

## Abbreviations

SM: Selective mutism
ASD: Autism spectrum disorder
DSM-5-TR: Diagnostic and statistical manual of mental disorders, fifth edition, text revision
PCIT-SM: Parent-child interaction therapy for selective mutism
IGBT-SM: Intensive group behavioral treatment for selective mutism
SPACE-SM: Specific parenting for anxious childhood emotions adapted for SM
BPT: Behavioral parent training
ABC analysis: Antecedent-behavior-consequence analysis
SM-BPT: SM-specific behavioral parent training program
ADHD: Attention-deficit/hyperactivity disorder
ID: Intellectual disability
SMQ-JP: Selective mutism questionnaire Japanese version
YCATS: Young children’s anxiety tendencies scale
CBCL: Child behavior check list 4–18 Japanese version
PSI: Parent stress index Japanese version
PNPS: Positive and negative parenting scale
GHQ-30: 30-item General Health Questionnaire 30 Japanese Version
SLD: Specific learning disorder
SNA: School non-attendance
